# Acute Life-Threatening Glycoprotein IIb/IIIa Inhibitor-Induced Thrombocytopenia Following Percutaneous Coronary Intervention (PCI): A Case Report and Review of the Literature

**DOI:** 10.7759/cureus.80907

**Published:** 2025-03-20

**Authors:** Baraa Souman, Cara Wyant, Zayd Parekh, Cyrus Shokoohi, Anisa Valina

**Affiliations:** 1 College of Osteopathic Medicine, Touro University Nevada, Henderson, USA; 2 Internal Medicine, Centennial Hills Hospital Medical Center, Las Vegas, USA

**Keywords:** adverse drug reaction (adr), anticoagulant-induced thrombocytopenia, critical care, drug-induced thrombocytopenia, glycoprotein iib/iiia inhibitors

## Abstract

Most patients who undergo percutaneous coronary intervention (PCI) to address coronary artery disease receive antiplatelets and anticoagulants to lower the risk of postoperative thrombotic events. Tirofiban, a glycoprotein IIb/IIIa inhibitor (GPI), has demonstrated remarkable efficacy in reducing morbidity and mortality rates in PCI postoperative care.

However, it is crucial to be vigilant about potential complications associated with tirofiban, particularly thrombocytopenia. Thrombocytopenia is a serious complication that requires close monitoring of the patients’ platelet count after initiation of the therapy. Regularly monitoring levels in two- to six-hour increments during the initial 24-48 hours after exposure can detect most cases of acute and potentially life-threatening thrombocytopenia. Prompt discontinuation of GPI and timely implementation of other supportive measures can help prevent further adverse events.

We present a case of a 70-year-old male who presented to the Emergency Department with chest pain. Following a thorough evaluation, the patient underwent angiography, during which stent placement was performed. Administration of tirofiban resulted in profound thrombocytopenia, with platelets decreasing to 1 g/L within 24 hours. Tirofiban was promptly withdrawn, and a platelet transfusion was initiated in order to stabilize the patient’s platelet level.

## Introduction

Patients who undergo coronary angiography and percutaneous coronary intervention (PCI) do not require glycoprotein IIb/IIIa inhibitor (GPI) therapy, particularly if the patient has received dual antiplatelet therapy (DAPT) with a potent P2Y12 inhibitor. GPIs are given to high-risk patients with a high thrombus burden, patients who have already received DAPT with ongoing ischemia, or are used intraoperatively for those who require an immediate coronary artery bypass graft [1,2].

Glycoprotein IIb/IIIa receptors are found on the surface of platelet membranes and serve to promote platelet aggregation by crosslinking fibrinogen [3]. For this reason, glycoprotein IIb/IIIa receptors have been one of the major targets in the management of high-risk acute coronary syndrome (ACS) and are used in conjunction with angioplasty, especially for bailout during thrombotic complications [4]. Although GPIs (abciximab, tirofiban, and eptifibatide) have been revolutionary in the management of ACS, drug-induced thrombocytopenia has been reported in approximately 0.1% to 2% of patients [3,5].

Tirofiban functions as a specific, competitive, non-peptide GPI that inhibits platelet aggregation in a dose-dependent manner when administered intravenously [2]. Evidence suggests tirofiban constructs a neoantigen determinant that can be recognized by host antibodies by altering the configuration of glycoprotein receptors on the membrane of platelets. These cells are subsequently targeted by the host immune system, resulting in increased platelet consumption and profound thrombocytopenia [3,6].

Here, we present a case of a 70-year-old patient who experienced severe acute thrombocytopenia after receiving therapy with tirofiban during and after PCI for non-ST-segment elevation myocardial infarction (NSTEMI).

## Case presentation

In August 2023, a 70-year-old man with a significant medical history of coronary artery disease, myocardial infarction status post-stenting, hypertension, hyperlipidemia, and HIV presented to the Emergency Department (ED) with left anterior chest pain, associated with radiation to the left neck and left upper extremity, nausea, vomiting, and diaphoresis for one hour prior to presentation. Prior to arrival, the patient was found to be in a hypotensive state with systolic blood pressure in the 60s; however, this was likely due to the patient reportedly taking two doses of nitroglycerin at the onset of his symptoms. The patient's blood pressure was stabilized during Emergency Medical Services (EMS) transport to the hospital with the administration of 1 L of normal saline by EMS. Physical examination in the ED was unremarkable, and the patient was hemodynamically stable. Initial EKG demonstrated a 1 mm elevation in V1, possible elevation in V2, and ST depression in lead II. While in the ED, the patient received amiodarone, aspirin, atorvastatin, heparin, and morphine, and was admitted for further observation. Cardiac catheterization and stent placement were performed two days after admission.

Investigations

Coronary angiography detected a 100% stenosis of the proximal-mid left anterior descending artery. A drug-eluting stent was placed, tirofiban and anticoagulation therapy were initiated, and the patient was admitted to the ICU for further management. Prior to catheterization, the patient’s platelets were found to be at 202 x 10^3^/mcL; however, repeat blood work less than 24 hours after the first tirofiban dose showed marked thrombocytopenia, with platelets at 1 x 10^3^/mcL. A review of the peripheral blood smear showed only a few rare platelets without platelet clumps, microangiopathy, or schistocytes. Immature myeloid cells were noted, as well as a left shift. Petechiae of the left thigh without evidence of hematoma or bruising was also discovered. The patient complained of a bowel movement with a moderate amount of hematochezia; however, this was determined to be due to hemorrhoids rather than a catastrophic GI bleed.

**Figure 1:**
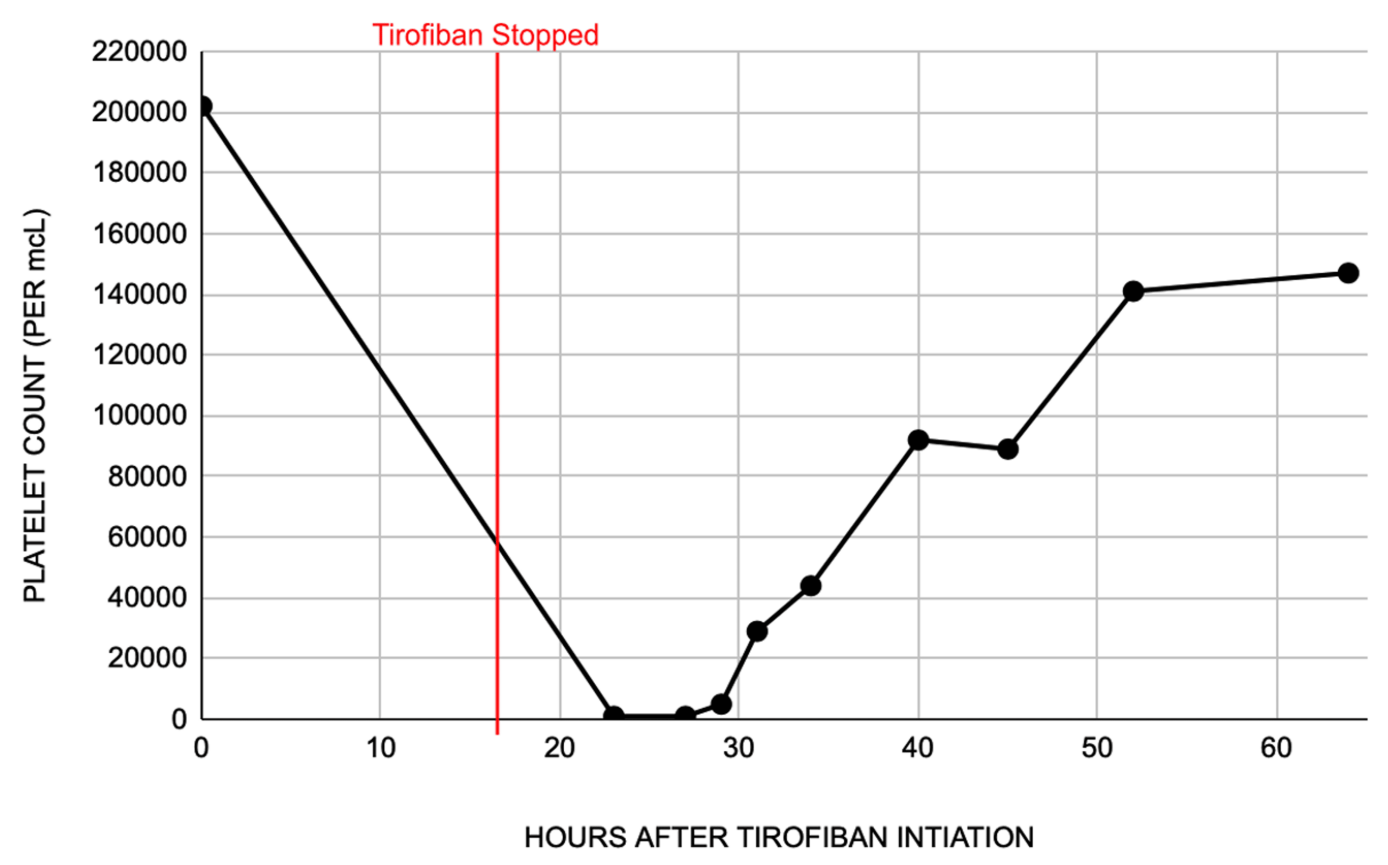
Platelet count at the initial administration of tirofiban, and immediately after the discontinuation of tirofiban.

Antiplatelets, aspirin and Brilinta, were immediately withheld, and four units of platelets were transfused. Within 24 hours of discontinuing tirofiban, the platelet count stabilized to 92 x 10^3^/mcL, and aspirin and Brilinta were resumed (Figure 1). The platelet count continued to trend upwards to 141 x 10^3^/mcL within 48 hours of discontinuation. The patient was downgraded to the intermediate care unit and ultimately discharged with a platelet count of 147 x 10^3^/mcL. The etiology of thrombocytopenia was concluded to be tirofiban-induced, as the platelet count was not affected by the antiplatelets aspirin and Brilinta when they were restarted, and other etiologies for thrombocytopenia were ruled out.

## Discussion

Glycoprotein IIb/IIIa receptors are found on the cell surface of platelet membranes and function by crosslinking fibrinogen, promoting platelet aggregation. For this reason, GPIs are used in conjunction with anticoagulants in the prevention of thrombotic events after PCI. Although the risk of future ischemic events, and related morbidity and mortality, are reduced [1], GPIs carry the risk of inducing potentially fatal immune-mediated thrombocytopenia.

The etiology of thrombocytopenia can fall into one of three categories: reduced production of platelets, increased consumption, or hemodilution. GPIs create a situation of increased consumption by immune-mediated degradation, often resulting in severe thrombocytopenia (platelet count <50 k/mm^3^) in 0.1%-2% of patients receiving therapy [3,5]. In this case, severe acute thrombocytopenia and anemia were discovered after the administration of tirofiban following a coronary intervention, to such a degree (1 x 10^3^ mcL) that has not been discussed in previous cases.

The possibility of heparin-induced thrombocytopenia (HIT) secondary to the administration of unfractionated heparin during the intervention was excluded, as the patient had been receiving heparin for over 48 hours prior to PCI and had stable platelet counts ranging from 202 to 237. HIT occurring within the first 24 hours of exposure may be seen in patients who have been previously exposed to heparin within the previous one to three months, which is not the case with this patient.

Given the potential for severe adverse effects, patients who receive GPI therapy should be monitored closely for platelet and hematocrit levels at regular two- to six-hour intervals. Other GPIs, such as abciximab and eptifibatide, have also been associated with thrombocytopenia [7]. Abciximab tends to have a higher incidence due to its irreversible receptor binding, whereas eptifibatide, like tirofiban, reversibly binds but has a different incidence profile [8]. Understanding these differences can be critical in drug selection and patient monitoring.

Once tirofiban has been identified as the cause of thrombocytopenia, the agent should be immediately discontinued, and the patient should be examined for signs and symptoms of active bleeding. In such cases of suspected hemorrhage, as in the patient in this report who was experiencing hematochezia with bowel movements, prompt evaluation of the anatomical source and immediate platelet transfusion should ensue [9]. If the patient does not have evidence of active bleeding, transfusion of platelets should be held due to the risk of stent thrombosis and infarction. Intravenous immunoglobulin (IVIG) treatment (400 mg/kg/day for five days) can also be used in cases of tirofiban-induced thrombocytopenia if the patient has evidence of active hemorrhage [2]. Since drug-dependent antibodies can persist in the patient’s serum for years, patients and providers should be educated on avoiding GPIs in the future.

Other common causes of acute thrombocytopenia, such as immune thrombocytopenic purpura (ITP) and thrombotic thrombocytopenic purpura (TTP), were briefly considered. However, these diagnoses were excluded based on clinical presentation, absence of characteristic laboratory findings, and the clear temporal association between thrombocytopenia onset and tirofiban administration [10]. Management of tirofiban-induced thrombocytopenia primarily involves immediate discontinuation of the drug. Current literature emphasizes careful monitoring of platelet counts, supportive therapy, and platelet transfusions in severe cases with active bleeding. Corticosteroids or IVIG can also be recommended in severe refractory cases, although evidence remains limited [11]. Importantly, an alternative antiplatelet or anticoagulation strategy may be necessary to ensure ongoing treatment of the underlying coronary syndrome while managing thrombocytopenia.

## Conclusions

Glycoprotein IIb/IIIa receptor antagonists, specifically tirofiban, have been proven effective in decreasing thrombotic complications after PCI. However, the threat of sudden, serious thrombocytopenia as a result of GPI use should be closely monitored and managed. Early discontinuation of tirofiban, in combination with other supportive care, can resolve the life-threatening event.

## References

[1] Karvouni E, Katritsis DG, Ioannidis JP (2003). Intravenous glycoprotein IIb/IIIa receptor antagonists reduce mortality after percutaneous coronary interventions. J Am Coll Cardiol.

[2] Gulati A, Tiwari A, Shetty V, Nwosu I, Khurana S (2021). Tirofiban: a rare cause of thrombocytopenia in a patient undergoing percutaneous coronary intervention. Cureus.

[3] Rahman N, Jafary FH (2010). Vanishing platelets: rapid and extreme tirofiban-induced thrombocytopenia after percutaneous coronary intervention for acute myocardial infarction. Tex Heart Inst J.

[4] Amsterdam EA, Wenger NK, Brindis RG (2014). 2014 AHA/ACC guideline for the management of patients with non-ST-elevation acute coronary syndromes: executive summary: a report of the American College of Cardiology/American Heart Association Task Force on practice guidelines. Circulation.

[5] Aster RH, Curtis BR, McFarland JG, Bougie DW (2009). Drug-induced immune thrombocytopenia: pathogenesis, diagnosis, and management. J Thromb Haemost.

[6] Yurtdaş M, Yaylali YT, Aladağ N, Ozdemir M, Atay MH (2014). Acute serious thrombocytopenia associated with intracoronary tirofiban use for primary angioplasty. Case Rep Med.

[7] Waller DG, Sampson AP (2018). Haemostasis. Medical Pharmacology and Therapeutics.

[8] Bansal AB, Sattar Y, Patel P (2025). Eptifibatide. StatPearls [Internet].

[9] Rawala MS, Ahmed AS, Posina K, Sundaram V (2020). Tirofiban induced thrombocytopenia: a rare but severe adverse effect. J Community Hosp Intern Med Perspect.

[10] Pietras NM, Gupta N, Justiz Vaillant AA (2025). Immune thrombocytopenia. StatPearls [Internet].

[11] Wang J, Zou D (2023). Tirofiban-induced thrombocytopenia. Ann Med.

